# Relapse of Diffuse Large B‐Cell Lymphoma as Painless Masses in the Abdominal Wall Muscles: A Rare Case Report

**DOI:** 10.1002/cnr2.70114

**Published:** 2025-01-06

**Authors:** Somar Mansour, Seif‐Aldin Abdul Rahman, Majd Mansour, Ali Afif, Raghad Hasan, Nader Abdullah, Zuheir Alshehabi

**Affiliations:** ^1^ Cancer Research Center Tishreen University Hospital Latakia Syria; ^2^ Faculty of Medicine Tishreen University Latakia Syria; ^3^ Department of Oncology Tishreen University Hospital Latakia Syria

**Keywords:** abdominal, DLBCL, lymphoma, muscle, painless

## Abstract

**Background:**

The most frequent type of non‐Hodgkin lymphoma (NHL) is diffuse large B‐cell lymphoma (DLBCL). Although lymph nodes are the most commonly affected organs compromising 70% of DLBCLs, only 5% of extranodal lymphomas represent skeletal muscle involvement. Specifically, abdominal wall muscle involvement is rare and there are only a few reported cases of DLBCL with this type of muscle involvement. Painful abdominal mass was the main presenting symptom in these reported cases.

**Case:**

We are reporting a relapsed DLBCL with abdominal wall muscle involvement in a 65‐year‐old male, presenting with a discomfort and heaviness sensation in the right iliac region with no associated pain.

**Conclusion:**

A rare case of DLBCL with recurrence in the abdominal wall muscles as painless masses was reported in this case report. To our knowledge, it is considered the fourth reported in the medical literature. It shows the importance of the diagnostic process that combines imaging with histological examination and immune stains for accurate diagnosis.

## 1 Introduction

The most frequent type of non‐Hodgkin lymphoma (NHL) is diffuse large B‐cell lymphoma (DLBCL) which is an aggressive B‐cell lymphoma, predominantly affecting older individuals [1]. DLBCL most commonly affects the lymph nodes but may occur in almost any organ system which partially reflects the variety of the B‐cell system. The involvement of soft tissues (especially skeletal muscles) is not a common finding and accounts for only 5% of extranodal NHLs. Specifically, the involvement of abdominal wall muscles is extremely rare [2].

DLBCL also varies by its response to treatment, but the current treatment standard is R‐CHOP protocol (rituximab, cyclophosphamide, doxorubicin, vincristine, and prednisone), with cure rates overriding 50%. There is no standard therapy for patients with relapsed lymphomas. However, there are some protocols [ex, R‐ICE (rituximab, ifosfamide, carboplatin, and etoposide)], which are proven to be well‐tolerated and good for patients with relapsed DLBCL [3].

## 2 Case Presentation

In December 2022, a 65‐year‐old male went to the Oncology Clinic at Tishreen University Hospital, Latakia, Syria, 3 months after completing his R‐CHOP chemotherapy protocol for DLBCL with a complaint of discomfort and heaviness sensation in the right iliac region. The patient is hypertensive, and his medical history was significant for systemic non‐Hodgkin's Lymphoma (DLBCL) with involvement of the cervical, axillary, and abdominal lymph nodes and involvement of bone marrow (stage 4), which was treated for 6 months with R‐CHOP protocol and showed complete response according to the radiological and clinical examination of the patient.

Physical examination revealed the presence of a palpable mass in the right iliac region. Laboratory tests revealed white blood cells of 6800 per microliter (normal: 4500 to 11 000 per microliter), red blood cells of 4.57 million per microliter (normal: 4.35 to 5.65 million per microliter), CRP of 20.1 mg/L (normal: 0–6 mg/L), and ESR of 5 mm/h (normal: 0–20 mm/h).

**FIGURE 1:**
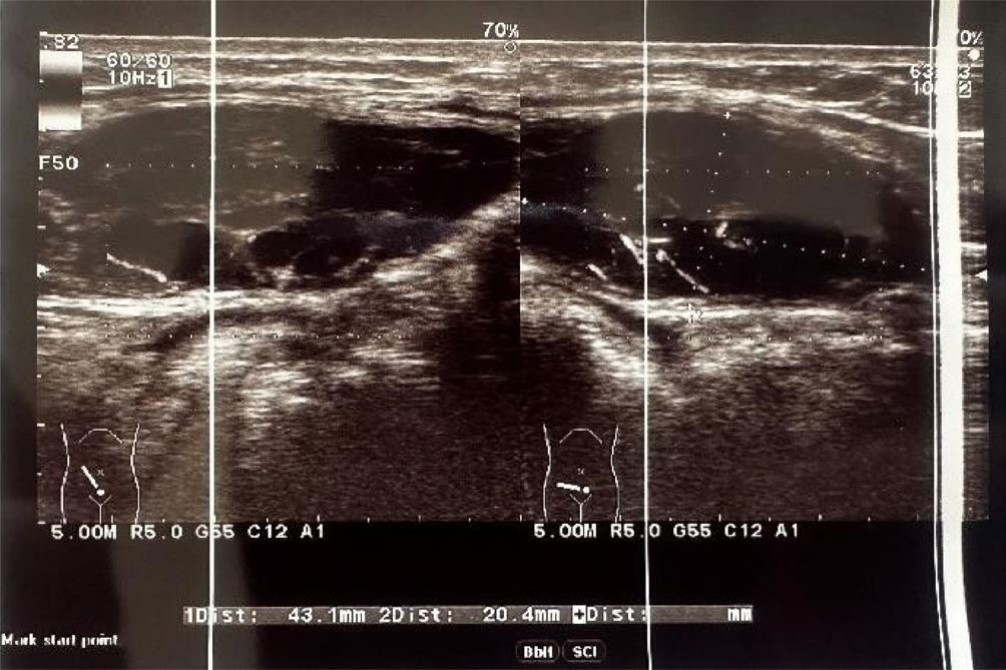
Ultrasonography of the abdominal wall showing multiple hypoechoic, disc‐shaped lesions.

Abdominopelvic ultrasonography was performed and showed three hypoechoic, disc‐shaped lesions above the muscle layer. The biggest mass was hypervascular and located inferomedially in the right iliac region measuring (50 × 20) mm and infiltrating the rectus abdominis muscle (Figure 1).

**FIGURE 2:**
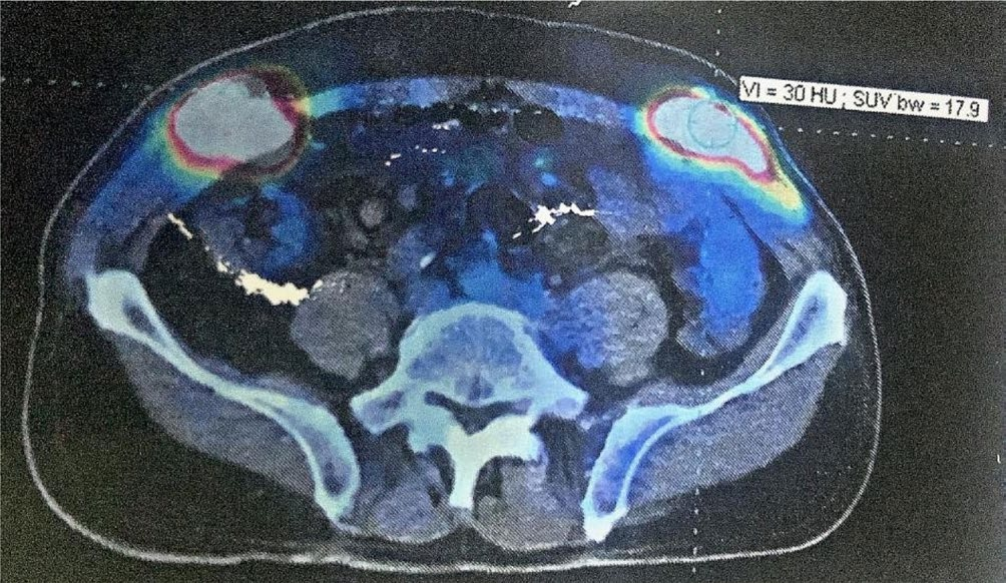
PET scan showing bilateral subcutaneous masses with high accumulation of radioactive material in the abdominal and pelvic wall with infiltration of the abdominal wall muscles.

A positron emission tomography (PET) scan was then ordered and showed multiple bilateral subcutaneous masses in the abdominal and pelvic wall; most of these lesions were located on the right side and were infiltrating the abdominal muscles (Rectus abdominis muscle). The largest mass, which has the highest accumulation of the radioactive material, was in the right iliac mass and measured (50 × 20) mm with a standardized uptake value (SUV) of 17.9 (Figure 2). Also, the PET scan showed masses with moderate uptake in the right peritoneum and the sigmoid wall as direct infiltration from abdominal muscles. These findings were consistent with a Stage 4 lymphoma (advanced stage), according to AJCC Cancer Staging Manual 2018, 8th edition.

**FIGURE 3:**
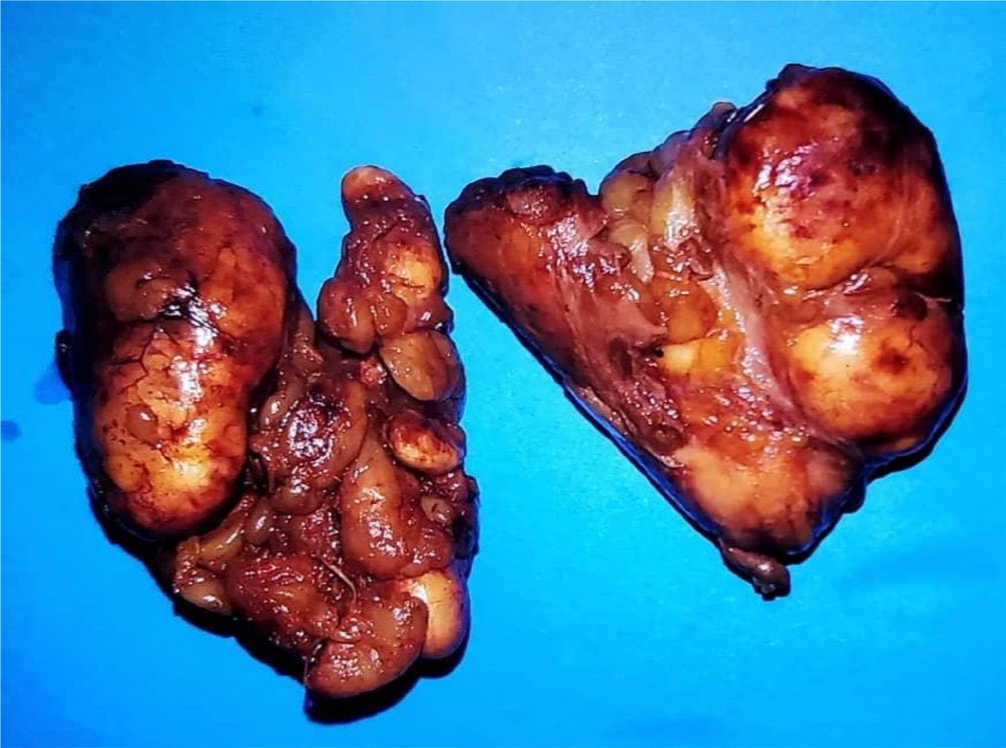
Gross inspection of the abdominal masses after the excisional biopsy.

**FIGURE 4:**
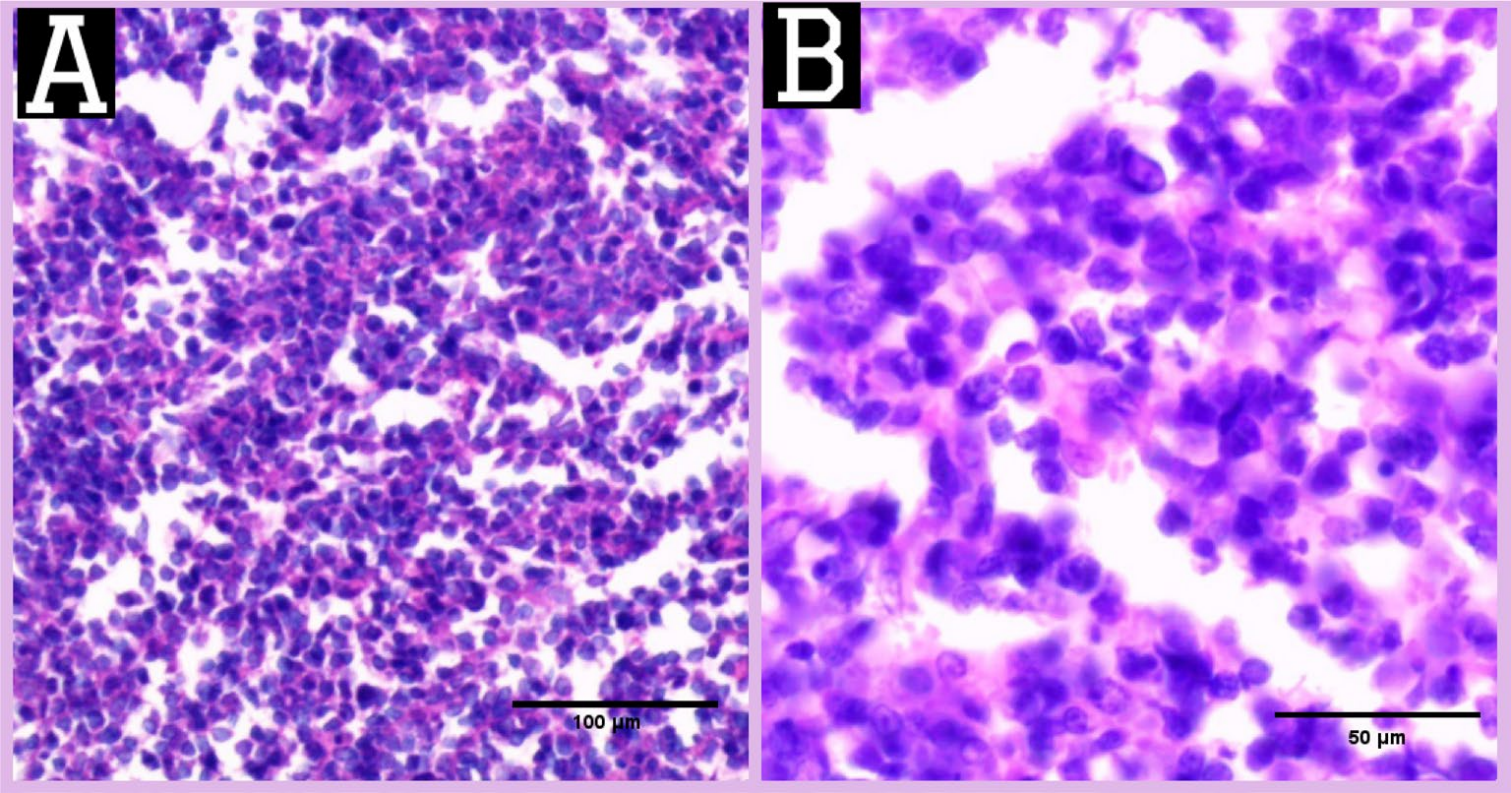
(A) Histopathology showing diffuse proliferation of atypical large lymphoid cells with a high nuclear‐to‐cytoplasmic ratio (H&E stain, ×200). (B) The large lymphoid neoplastic cells with prominent nucleoli (H&E stain × 400).

**FIGURE 5:**
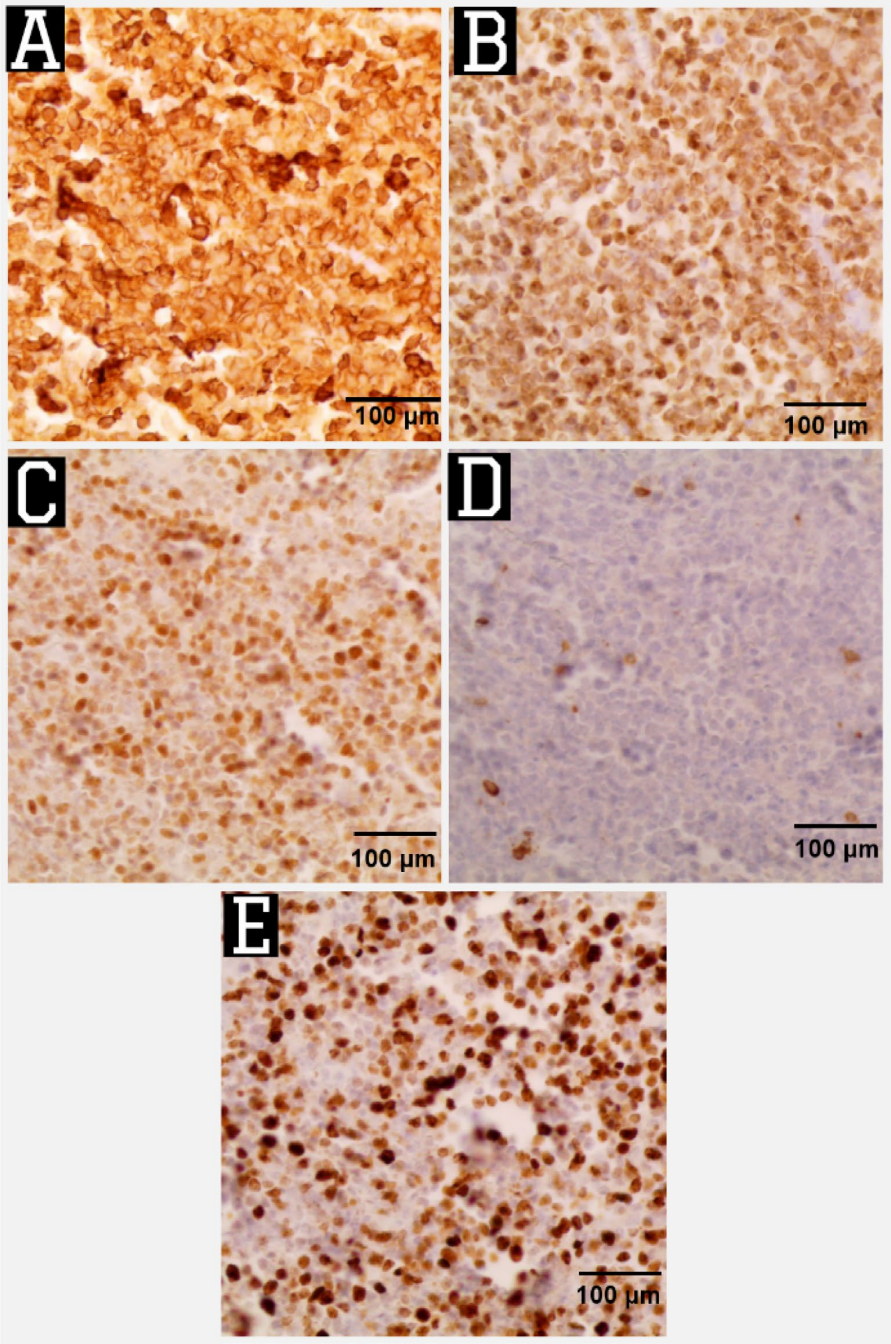
Immune stains show CD20 positivity (×100) [A], BCL2 positivity (×100) [B], BCL6 positivity (×100) [C], CD5 negativity (×100) [D], and Ki67: High rate ~ 80% (×100) [E].

Therefore, excisional biopsies were taken from the abdominal wall lesions and pathological findings showed soft to rubbery gray‐yellowish masses that consisted of large atypical lymphocytes with a high nuclear‐to‐cytoplasmic ratio (Figures 3 and 4). Subsequent immunohistochemistry studies showed positivity for CD20, BCL2, and BCL6 with negativity for CD5 and CD3. KI67 was approximately 80% (Figure 5). These findings were consistent with the recurrence of DLBCL.

Based on these findings, the patient was treated with R‐ICE chemotherapy protocol for 4 months which led to a decrease in the size of the abdominal lesions with no evidence of disease dissemination 3 months after completing the treatment. Unfortunately, more long‐term follow‐up could not be done as the patient has traveled out of our country to complete the treatment in another hospital due to the limitations in our hospital and the unavailability of autologous bone marrow transplantation.

## 3 Timeline

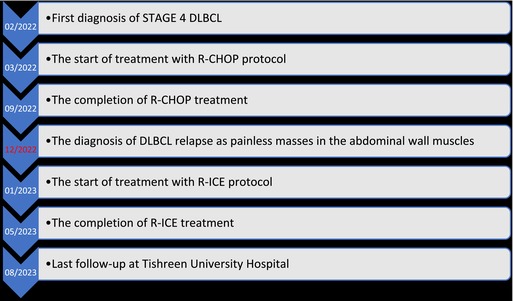

## 4 Discussion

DLBCL, which is the most common type of NHL, represents 30%–40% of NHL cases [4]. Although lymph nodes are the most commonly affected organs compromising 70% of DLBCLs, involvement of skeletal muscle is uncommon and accounts for only 5% of extranodal lymphomas. The extremities are the most common site of skeletal muscle lymphoma [2, 4].

According to Hatem and M. Bogusz's study in 2016, 61 of 86 patients (70.9%) in the literature with skeletal muscle lymphoma had DLBCL and only 2 of the 86 patients (2.3%) had abdominal muscle involvement as we found in our case [1]. After searching the literature, we found that only one DLBCL‐reported case after 2016 has presented with multiple masses involving abdominal wall muscles [2].

The complaint in all the three reported cases in the literature with abdominal muscle involvement was local pain in the abdominal wall, whereas in our case, the masses were painless and the patient complaint was only lower abdominal heaviness sensation especially when leaning forward which makes our case the first in the literature as a painless DLBCL with abdominal muscle involvement [2, 5, 6].

In medical practice, the primary imaging modality used to evaluate abdominal pain or masses is ultrasonography. However, when it comes to lymphomas affecting muscles, the features observed on ultrasonography are not specific and vary widely. These features may include the presence of hypoechoic solid mass with irregular or poorly defined borders [2].

In addition, a PET scan is a primary clinical imaging tool for staging, therapy evaluation, follow‐up, and assessment of patients with lymphoma. As we reported in our case, a PET scan played an essential role in the diagnosis of disease recurrence in the abdominal and pelvic walls [7].

Establishing the diagnosis is based on the histopathological and immunohistochemical evaluation. Microscopically, DLBCL is defined by diffuse infiltration of large atypical B lymphoid cells with large prominent nucleoli [8]. In addition, DLBCL shows a wide cytologic spectrum. Three frequent morphologic forms have been described, referred to as immunoblastic, centroblastic, and anaplastic variants [9].

Immunohistochemistry (IHC) is also an important tool used to type and confirmation of the characteristics of the tumor and detect its origin when presenting in unusual sites, especially in lymphomas. Also, determining the factors of prognosis in some subtypes of non‐Hodgkin's lymphoma is important [10]. DLBCL diagnosis is supported by the presence of (BCL2 expression, Ki‐67 rate < 90%, no cytogenetic evidence of MYC translocation, and BCL2 and/or BCL6 rearrangement). As we found in our case, IHC showed (Ki‐67 rate ~ 80%) and positivity of CD20, BCL2, and BCL6 with negativity for CD5 and CD3, confirming the diagnosis of recurrent DLBCL [11].

The standard protocol for the management of DLBCL is a combination of chemotherapy and immunotherapy, known as R‐CHOP with or without radiation therapy. However, only 60% of patients are cured with this therapy. Patients with localized lesions have also seen success with the approach of surgical excision followed by chemotherapy. The presentation in unusual sites may require different treatment protocols [1, 7, 12]. The number of elderly patients with relapsed lymphomas is increasing, and there is no standard treatment for these patients. Therefore, R‐ICE is a protocol that is considered to be a good and reasonable option for elderly patients with DLBCL which we used to treat our patient. It led to a decrease in the size of the abdominal lesions with no evidence of disease dissemination 3 months after completing the treatment [3].

## 5 Conclusion

DLBCL with skeletal muscle involvement is rare, especially when affecting abdominal wall muscles. In this case, we report a rare case of painless, relapsed DLBCL involving the muscles and subcutaneous tissue of the abdominal wall and highlight the steps of diagnostic and therapeutic approaches This case shows the importance of considering DLBCL in differential diagnoses of abdominal masses, even when painless, and highlights the need for comprehensive diagnostic approaches combining imaging with histological assessments to accurately diagnose. To our knowledge, we are presenting painless abdominal wall lymphoma with muscle involvement, which will greatly benefit the medical literature.

## Author Contributions

**Somar Mansour:** writing original draft – review and editing. **Seif‐Aldin Abdul Rahman:** review and editing. **Majd Mansour:** writing the original draft. **Ali Afif:** writing the original draft. **Raghad Hasan:** writing the original draft. **Nader Abdullah:** review and editing. **Zuheir Alshehabi:** supervision – review and final editing.

## Consent

Written informed consent was obtained from the patient for publication of this case report.

## Conflicts of Interest

The authors declare no conflicts of interest.

## Data Availability

Data and material are available upon reasonable request from the corresponding author.
